# Japanese Practicing Physicians' Relationships with Pharmaceutical Representatives: A National Survey

**DOI:** 10.1371/journal.pone.0012193

**Published:** 2010-08-13

**Authors:** Sayaka Saito, Kei Mukohara, Seiji Bito

**Affiliations:** 1 Department of General Medicine and Primary Care, Tsukuba Medical Center Hospital, Ibaraki, Japan; 2 Department of General Internal Medicine, National Hospital Organization Nagasaki Medical Center, Nagasaki, Japan; 3 Division of Clinical Epidemiology, National Hospital Organization Tokyo Medical Center, Tokyo, Japan; University of British Columbia, Canada

## Abstract

**Background:**

Previous surveys on the relationship between physicians and pharmaceutical representatives (PRs) have been of limited quality. The purpose of our survey of practicing physicians in Japan was to assess the extent of their involvement in pharmaceutical promotional activities, physician characteristics that predict such involvement, attitudes toward relationships with PRs, correlations between the extent of involvement and attitudes, and differences in the extent of involvement according to self-reported prescribing behaviors.

**Methods and Findings:**

From January to March 2008, we conducted a national survey of 2621 practicing physicians in seven specialties: internal medicine, general surgery, orthopedic surgery, pediatrics, obstetrics-gynecology, psychiatry, and ophthalmology. The response rate was 54%. Most physicians met with PRs (98%), received drug samples (85%) and stationery (96%), and participated in industry-sponsored continuing medical education (CME) events at the workplace (80%) and outside the workplace (93%). Half accepted meals outside the workplace (49%) and financial subsidies to attend CME events (49%). Rules at the workplace banning both meetings with PRs and gifts predicted less involvement of physicians in promotional activities. Physicians valued information from PRs. They believed that they were unlikely to be influenced by promotional activities, but that their colleagues were more susceptible to such influence than themselves. They were divided about the appropriateness of low-value gifts. The extent of physician involvement in promotional activities was positively correlated with the attitudes that PRs are a valuable source of information and that gifts are appropriate. The extent of such involvement was higher among physicians who prefer to ask PRs for information when a new medication becomes available, physicians who are not satisfied with patient encounters ending only with advice, and physicians who prefer to prescribe brand-name medications.

**Conclusions:**

Involvement in pharmaceutical promotional activities is widespread among practicing physicians in Japan. The extent of such involvement varies according to certain physician characteristics. As a group, they are at risk for influence by promotional activities.

## Introduction

In the past few decades, the relationship between physicians and the pharmaceutical industry has been one of the most controversial issues in medicine. Pharmaceutical representatives (PRs) have direct contact with physicians and play a key role in the promotional activities of the pharmaceutical industry. Previous surveys have examined conflicts of interest in physicians' relationships with PRs. These studies found that many physicians met PRs frequently [1]–[11] and were involved in a variety of promotional activities [3], [5], [6], [9], [11]–[16]. Physicians variably rated the informational value of PRs in medical education [1]–[3], [5]–[8], [10], [12], [15]–[18]. Their beliefs about the influence of interactions with PRs on prescribing behaviors were mixed [1], [3], [5]–[8], [10], [13], [14], [16], [18], [19]. They believed that their colleagues were more likely to be influenced than themselves [13], [14], [16], [19]. Physicians tended to believe that it was appropriate to receive inexpensive gifts [2], [6], [8], [14], [16], [18], [19]. Physicians who met with PRs frequently were more likely to prescribe medications that are not clinically indicated [20], and to rely less on information from published research findings when a new medication becomes available [20]. However, the quality of these surveys was limited because of their small sample sizes [1]–[10], [12]–[19], the use of accessible and convenience samples such as residents and faculty members in academic medical centers [1]–[3], [5], [7], [9], [14]–[16], [18], and the lack of comprehensive exploration of the relationships among physician characteristics, involvement in promotional activities, attitudes, and prescribing behaviors [11], [20].

We conducted a national survey of practicing physicians in Japan. The purpose of the survey was to answer five questions regarding physician involvement in promotional activities, by which we mean meeting with PRs, receiving gifts, and participating in promotional events. First, what is the extent of physician involvement in promotional activities? Second, what physician characteristics predict such involvement? Third, what attitudes do physicians have toward relationships with PRs? Fourth, are there correlations between the extent of the involvement and their attitudes? Finally, are there differences in the extent of the involvement according to self-reported prescribing behaviors?

## Methods

### Survey Design

The study was a cross-sectional survey that used a 26-item, 4-page, anonymous, self-administered questionnaire. The questionnaire was developed based on literature review [1], [5], [11], [14], [20] and discussions between two authors (SS, KM). The questionnaire sought characteristics of the respondents, frequency of involvement in pharmaceutical promotional activities, attitudes toward relationships with PRs, and self-reported prescribing behaviors (File S1). The questionnaire was not pretested for its validity and reliability before being administered in the study. The ethics committee at Kawasaki Saiwai Hospital approved the survey protocol.

#### Characteristics of the respondents

In addition to sex, years in practice, specialty and practice setting, we asked how much opportunity respondents had had to learn physician-industry relationships and critical appraisal skills on a five-point scale (none, little, a little, some, substantial) and whether their workplaces had rules banning meetings with PRs, gifts, or both (File S1).

#### Involvement in promotional activities

Respondents were asked to report the frequency of meetings with PRs and receiving or participating in various types of gifts or promotional events on a five-point scale: never = 0, less than once a month = 1, two to three times a month = 2.5, once a week = 4, two to three times a week = 10, nearly every day = 20 (File S1). Responses to these questions were summed to create a “Promotional Activity Index Score” (range 0–140). The score expressed the frequency of meeting with PRs and receiving gifts or participating in the promotional events listed in our survey in a typical four-week period.

#### Attitudes toward relationships with PRs

We asked respondents to report their degree of agreement with a series of eight statements about relationships with PRs on a five-point scale: agree, somewhat agree, neutral, somewhat disagree, and disagree (File S1). Principal component factor analysis for these eight statements extracted three factors (File S2). They consisted of three items (statement 3a, 3b, 3c) for which agreement suggests that respondents believe that PRs are a valuable source of information (‘Informational Value Scale’), three items (statement 3d, 3e, 3f) for which disagreement suggests that they are immune from influence by interactions with PRs (‘Immunity Scale’), and two items (statement 3g, 3h) for which agreement suggests that they believe receiving gifts is appropriate (‘Appropriateness Scale’). The points for each of these three scales were converted to a summary score ranging from 0 to 1.0 (File S3). A score of 0 was assigned to physician perception of PRs as having minimal informational value, themselves as minimally immune to promotion, and gifts as totally inappropriate. A score of 1.0 was assigned to physician perception of PRs as having maximal informational value, themselves as maximally immune to promotion, and gifts as totally appropriate.

#### Prescribing behaviors

Respondents were asked about four questions relating to their prescribing behaviors (File S1).

### Survey Sample

The target population was practicing physicians both in office and hospital settings in Japan. According to a national survey of physicians' demographic characteristics [21], the total number of physicians in Japan in 2004 was 270,371, of which 92,985 physicians worked in office settings and 163,683 physicians worked in hospital settings. We were unable to sample randomly because there is no complete up-to-date registry of all physicians in Japan. We used internet search engines to identify our survey participants. In order to ensure representativeness of our sample, we pre-specified inclusion criteria based on a national survey of physicians as follows [21]: We included internists, general surgeons, orthopedic surgeons, pediatricians, obstetrician-gynecologists, psychiatrists, and ophthalmologists because these were the seven most numerous specialties in Japan. Equal numbers of office-based and hospital-based physicians were included because the national survey showed the ratio of the number of office-based and hospital-based practicing physicians was approximately one to one [21]. We included 4 office-based and 4 hospital-based physicians practicing in each of 7 specialties for all 47 Japanese prefectures. We excluded those who worked in academic medical centers, were retired or on leave, or were in administrative positions in hospitals.

### Survey Administration

The surveys were administered from January to March 2008. To build participant expectations, a pre-notification postcard was sent to all participants. Five days after sending the pre-notification postcard, we sent every participant a cover letter, a questionnaire, a self-addressed postcard with an individual participant's name, a stamped reply envelope, and a 500 yen (approximately US$5) prepaid gift card for use at bookstores as an incentive. The cover letter explained the purpose of the study, the voluntary nature of participation, and the confidentiality of responses. We asked participants to return the completed questionnaire separately from the self-addressed postcard. The postcard allowed us to track non-respondents while maintaining the anonymity of respondents. Non-respondents received up to two reminders along with copies of the original questionnaire at two-week intervals. We made clear to participants that the study was sponsored by the Ministry of Education, Culture, Sports, Science and Technology (MEXT) in order to assure them of its credibility.

A questionnaire was considered to be evaluable when it was returned by the pre-specified deadline (March 17, 2008) and had complete information for 80% or more of all 26 items. Multiple responses to a question for which a single response was appropriate were considered to be no answer and not evaluable. In order to ensure completeness and accuracy of data entry, two of the authors (SS, KM) independently entered the data into Microsoft Excel. Then two datasets were compared by the same two authors independently. Errors in data entry were corrected. Disagreements were resolved through discussion.

### Statistical Analysis

Multivariate logistic regression models were used to assess associations between physician characteristics and involvement in promotional activities. We performed pairwise comparisons of each specialty with every other specialty. Bonferroni corrections were used to adjust for multiple comparisons (a total of 29). A *p* value of less than 0.0017 (0.05 divided by 29) was considered to indicate statistically significant differences between specialties. Wilcoxon's signed rank test was used to compare perceptions about informational value of PRs for new vs. well-established medications and perceptions about a participant's own immunity vs. others' immunity to influence. Pearson product-moment correlations were used to characterize relationships between Promotional Activity Index Score and each of three attitudinal scores. *T*-tests were used to compare Promotional Activity Index Scores of two groups of physicians with different self-reported prescribing behaviors. For multivariate logistic regression analyses, odds ratios with 95% confidence intervals were calculated. For all of the statistical analyses except for the analysis of multiple comparisons between specialties, *p* values of less than 0.05 were considered to be statistically significant. All *p* values were 2-tailed. SPSS, version 16.0 was used for all statistical analyses.

## Results

### Study Population

We identified a total of 2632 physicians via an internet search based on our pre-specified inclusion and exclusion criteria, and we sent the survey questionnaire to all of them. Of the 2632 physicians, 11 were ineligible to participate in the survey because they were not providing patient care, out of the country, or practicing in a specialty not included in the survey. We were unable to contact 34 physicians, and 22 declined to answer. Thirty-one answer sheets were incomplete. Of the 2621 judged to be either eligible or of unknown eligibility, 1411 physicians completed the survey, for a response rate of 54%. We referred to the standard definitions of the American Association for Public Opinion Research to calculate the response rate [22].

The characteristics of the respondents are shown in Table 1. The proportions of women to men and of office-based to hospital-based physicians among our respondents were slightly different from the national proportions in 2004 (23% vs. 17% women and 58% vs. 45% office-based physicians, respectively) [21]. The distribution of years in practice approximated the national distribution, and the distribution among specialties was not skewed toward one particular group. Twenty-six percent of respondents reported having had opportunities to learn physician-industry relationships and 47% reported having learned critical appraisal skills. Seventy-six percent reported that their workplaces did not have local rules banning meetings with PRs or gifts. Only five percent reported that their workplaces had rules banning both meeting with PRs and gifts.

**Table 1:** Characteristics of 1411 survey respondents.

Characteristic	Respondents	
	No./Total No.	Percentage*
Sex		
Male	1084/1410	77
Female	326/1410	23
No. of years in practice		
10 yr. or less	339/1410	24
11–20 yr.	488/1410	35
21–30 yr.	428/1410	30
31 yr. or more	155/1410	11
Specialty		
Internal Medicine	214/1409	15
General surgery	181/1409	13
Orthopedic surgery	177/1409	13
Pediatrics	221/1409	16
Obstetrics/gynecology	210/1409	15
Psychiatry	197/1409	14
Ophthalmology	209/1409	15
Practice setting		
Office	822/1410	58
Hospital	588/1410	42
Having had opportunities to learn physician-industry relationships**		
Yes	370/1406	26
No	1036/1406	74
Having had opportunities to learn critical appraisal skills**		
Yes	666/1406	47
No	740/1406	53
Rules banning meetings with PRs and/or gifts at the workplace		
Yes Rules banning both meetings with PRs and gifts	63/1391	5
Rules banning meetings with PRs, not gifts	54/1391	4
Rules banning gifts, not meetings with PRs	217/1391	16
No	1057/1391	76

Abbreviation: PR, pharmaceutical representative.

*Percentages may not add up to exactly 100% due to rounding.

**Asked on five-point Likert scale (none, little, a little, some, substantial). ‘Yes’ was defined as ‘a little, some, or substantial’. ‘No’ was defined as ‘none, or little.’

### The extent of involvement in promotional activities

Table 2 shows respondents' involvement in promotional activities. Most physicians met with PRs, received drug samples and stationery, and participated in industry-sponsored continuing medical education (CME) events at and outside the workplace. Half accepted meals outside the workplace and financial subsidies to attend CME events. On average, they met with PRs seven times per month and received gifts or participated in events once to twice per month. Internists met with PRs most frequently (ten times per month), followed by general surgeons and orthopedic surgeons (eight times per month), pediatricians and ophthalmologists (seven times per month), psychiatrists (six times per month), and obstetrician-gynecologists (five times per month).

**Table 2:** Physician involvement in various types of pharmaceutical promotional activities.

Type of pharmaceutical promotional activities	Number of respondents who meet with PRs, receive gifts, or participate in events	Frequency of involvement per month
	Number/Total number (%)	Mean (SD)
Meetings with PRs	1383/1407 (98)	7.1 (5.3)
Drug samples	1190/1408 (85)	1.4 (1.5)
Stationery such as pens and notepads	1347/1408 (96)	2.2 (2.2)
Meals outside the workplace	697/1410 (49)	0.6 (0.8)
Industry-sponsored CME events at the workplace	1132/1410 (80)	1.1 (1.0)
Industry-sponsored CME events outside the workplace	1315/1408 (93)	1.2 (0.9)
Financial subsidies to attend CME events	696/1410 (49)	0.6 (0.7)

Abbreviations: PR, pharmaceutical representative; CME, continuing medical education.

### Physician characteristics that predict involvement in specific types of promotional activities

Multiple logistic regression analyses identified independent predictors of physician involvement in specific types of promotional activities (Table S1).

#### Meetings with PRs

Physicians whose workplaces banned both meeting with PRs and gifts were less likely to meet with PRs than those whose workplaces without such rules. No other predictors were identified.

#### Drug samples

Physicians in practice for 21 years or more were less likely to receive drug samples than those in practice for 20 years or less. Physicians whose workplaces banned both meetings with PRs and gifts were less likely to receive drug samples. Internists, orthopedic surgeons, pediatricians, and ophthalmologists were more likely than psychiatrists to receive drug samples.

#### Stationery such as pens and notepads

Physicians whose workplace banned both meetings with PRs and gifts were less likely to receive stationery. No other predictors were identified.

#### Meals outside the workplace

Female (vs. male) physicians, physicians in practice 21 years or more (vs. 20 years or less), and hospital-based (vs. office-based) physicians, and physicians whose workplaces banned both meetings with PRs and gifts or banned gifts only were less likely to accept meals outside the workplace. Orthopedic surgeons were more likely to accept such meals than obstetrician-gynecologists and ophthalmologists.

#### Industry-sponsored CME events at the workplace

Female (vs. male) and hospital-based (vs. office-based) physicians were more likely to participate in industry-sponsored CME events at the workplace. Obstetrician-gynecologists were less likely than internists to participate in such events.

#### Industry-sponsored CME events outside the workplace

Physicians whose workplaces banned both meetings with PRs and gifts were less likely to participate in industry-sponsored CME events outside the workplace than those whose workplaces had no such rules. No other predictors were identified.

#### Financial subsidies to attend CME events

Obstetrician-gynecologists and ophthalmologists were less likely to receive financial subsidies to attend CME events than the other specialists. Physicians whose workplaces banned both meetings with PRs and gifts or banned gifts only were less likely to receive such subsidies.

### Attitudes toward relationships with PRs

**Figure 1:**
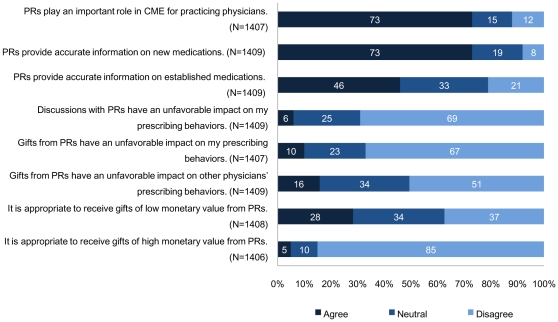
Attitudes toward relationships with pharmaceutical representatives. Abbreviations: PR, pharmaceutical representative; CME, continuing medical education.

For graphical presentation, answers were aggregated into agree, neutral, and disagree (Figure 1). Informational Value Scores were calculated for the 1405 physicians (99.6%) who completed all three items. Cronbach's alpha for Informational Value Scores was 0.75. The mean Informational Value Score was 0.66 (SD, 0.20; range 0–1.0), indicating that physicians were likely to believe that PRs are a valuable source of information. Their perceptions about PRs' informational value for new medications were higher than that for well-established ones (*p*<.001). Immunity Scores were calculated for 1405 physicians (99.6%) who completed all three items. Cronbach's alpha for Immunity Scores was 0.75. The mean Immunity Score was 0.69 (SD, 0.20; range 0–1.0), indicating that they believed that they were immune from having their practice influenced by discussions with and gifts from PRs. While most thought that their own and their colleagues' prescribing behaviors were not likely to be influenced, they believed that their colleagues were more likely to be influenced than themselves (*p*<.001). Appropriateness Scores were calculated for 1405 physicians (99.6%) who completed both items. Cronbach's alpha for Appropriateness Scores was 0.65. The mean Appropriateness Score was 0.30 (SD, 0.23; range 0–1.0), indicating that they thought gifts from PRs were more likely to be inappropriate than to be appropriate. Physicians thought that gifts of low value were more appropriate than those of high value. They were divided about the appropriateness of low-value gifts (28% were in favor vs. 37% opposed).

### Correlations between the extent of involvement in promotional activities and attitudes

There were modest but statistically significant positive correlations between Promotional Activity Index Score and Informational Value Score (*r* = 0.161; *p*<.001) and between Promotional Activity Index Score and Appropriateness Score (*r* = 0.158; *p*<.001). There was no correlation between Promotional Activity Index Score and Immunity Score (*r* = 0.038; *p* = .159).

### Differences in the extent of involvement in promotional activities according to self-reported prescribing behaviors

The extent of involvement in promotional activities was greater among physicians who prefer to ask PR for information when a new medication becomes available, physicians who are not satisfied with the patient encounter ending only with advice, and physicians who prefer to prescribe brand-name medications when generic options are available (Table 3). There was no statistically significant difference in the extent of involvement according to such self-reported prescribing behaviors as (1) a greater willingness to prescribe a new medication to a few patients and monitor outcomes when it becomes available or (2) to agree with patients' requests to prescribe medications that are not clinically indicated.

**Table 3:** Comparisons of Promotional Activity Index Scores according to prescribing behaviors.

Prescribing behavior		Number (%) of respondents*	Promotional Activity Index Score** (range, 0–140)	
			Mean (SD)	*p*-value
When a new medication becomes available, what I do most commonly first is:***	Ask PRs for information	578/1387 (42)	15.0 (8.4)	.002
	Seek published literature for effectiveness or ask colleagues or specialists	758/1387 (55)	13.6 (8.5)	
When faced with a patient who expects a prescription (which is not clinically indicated) my usual response is to:	Prescribe readily or reluctantly	306/1387 (22)	14.8 (8.7)	.139
	Not prescribe	1081/1387 (78)	14.0 (8.4)	
I feel that a patient consultation that ends with me giving advice only is	Unsatisfactory or somewhat satisfactory	670/1388 (48)	14.9 (8.8)	.003
	Satisfactory	718/1388 (52)	13.5 (8.1)	
When generic options are available, I think we should prescribe:	Brand name medications	322/1396 (23)	15.3 (9.5)	.005
	Neutral or generic medications	1074/1396 (77)	13.8 (8.0)	

Abbreviation: PR, pharmaceutical representative.

*Percentages may not add up to exactly 100% due to rounding.

**Promotional Activity Index Score expresses the frequency of meeting with PRs, receiving gifts, and participating in promotional events listed in our survey in a typical four-week period.

***We also compared promotional activity index scores between those who answered ‘use on a few patients and monitor’ and ‘seek published literature for effectiveness or ask colleagues or specialists’ and found no difference (13.3 [7.4] vs. 13.6 [8.5]; p = .803).

## Discussion

Most physicians in our survey were involved in promotional activities. The extent of such involvement varied according to certain physician characteristics. Many physicians valued PRs as a source of information and believed they were unlikely to be influenced by promotional activities. They were divided about the appropriateness of low-value gifts. The extent of involvement was positively correlated with the attitudes that PRs are a valuable source of information and that gifts are appropriate. The extent of such involvement was higher among physicians who prefer to ask PRs for information when a new medication becomes available, physicians who are not satisfied with patient encounters ending only with advice, and physicians who prefer to prescribe brand-name medications.

Practicing physicians in Japan met with PRs five to ten times per month depending on specialty. These findings are similar to those of a recent national survey in the US, which found that internists met with PRs ten times per month, pediatricians eight times per month, and surgeons four times per month [11]. In our survey, Japanese physicians received various types of gifts or participated in promotional CME events once to twice per month, which is likely to be an underestimate because the maximum number that could be indicated in our survey was six gifts or events. Physicians might be receiving gifts or participating in events beyond this number.

Most physicians attended industry-sponsored CME events outside the workplace. One explanation for this finding may be that professional medical associations require physicians to earn CME credits and the pharmaceutical industry sponsors educational programs offering credits.

In our survey, physician-reported educational experiences in physician-industry relationships and critical appraisal skills did not predict the frequency of involvement in promotional activities. This may be due to inaccurate self-assessment of respondents' personal levels of educational experiences.

Multivariate logistic regression models identified independent predictors of physician involvement in types of promotional activities. Of note, while bans on gifts only lessened the likelihood of physicians' accepting meals outside the workplace and financial subsidies to attend CME events, bans on both meetings with PRs and gifts lessened the likelihood of their meetings with PRs, receiving drug samples, receiving stationery, and participating in industry-sponsored CME events outside the workplace. Effective rules banning not only gifts from PRs but also meeting with PRs may be needed to significantly limit the extent of physician involvement in promotional activities.

Many respondents valued PRs as sources of information. They perceived that the informational value of PRs was higher for new medications than for well-established ones. One explanation of this finding may be that physicians can obtain information about well-established medications from various sources, while they can find much less information about new medications. Another explanation may be that PRs provide more information about new medications for promotional purposes. As in other studies [13], [14], [16], [19], physicians believed that their colleagues were more likely to be influenced by promotional activities than themselves. These findings are consistent with a line of social science research which shows that individuals are susceptible to an unconscious and unintentional “self-serving bias”: judgments of fairness are biased in favor of self-interests [23]. Physicians were divided about the appropriateness of accepting low-value gifts. This contrasts with the findings in other surveys that the majority of respondents considered low-value gifts as appropriate or not ethically problematic. There were modest but statistically significant correlations between the extent of involvement in promotional activities and attitudes about the informational value of PRs and the appropriateness of gifts. This finding is consistent with a national survey of medical students in the US [24], although measurements of promotional activities and attitudes were not identical in both surveys. The mean Promotional Activity Index Scores were slightly but statistically significantly higher among physicians who prefer to ask PRs for information about a new medication, those who are not satisfied with patient encounters ending only with advice, and those who prefer to prescribe brand-name medications. These findings suggest but do not prove that promotional activities have a modest impact on physicians' prescribing behaviors. Ideally, intervention studies are needed to prove whether and to what extent promotional activities affect physicians' prescribing behaviors.

Our survey has several limitations. First, the fact that our survey sample was not selected randomly might have compromised its representativeness. In order to overcome this limitation, we devised our sampling method based on national demographic data of all registered physicians in 2004 [21]. The background information of our respondent - such as sex, years in practice, and practice settings - did not significantly differ from national data. Second, despite the use of the standard techniques for improving responses to mailed surveys, the overall response rate did not reach 60%, generally considered to be necessary for the validity of a survey [25]. Third, the respondents might have reported attitudes and behaviors that are socially desirable, although we attempted to minimize this bias by ensuring the anonymity of the respondents. Fourth, the cross-sectional nature of the survey does not allow us to infer causality of the associations. Fifth, our results may not be generalizable to physicians in countries other than Japan because of differences in the context. The Japanese healthcare system has distinctive characteristics such as coverage of all citizens by universal health insurance system, a lack of a primary care system, few certified generalist physicians, and guaranteed access to specialist physicians without a referral. There is much less public discussion about physician-industry relationships both in the lay media and the medical literature as compared with the US or other relevant countries. There is currently no code of ethics promulgated by industry, professional societies, or the government of Japan concretely defining appropriate interactions between physicians and pharmaceutical industry or prohibiting inappropriate interactions. There are few formal curricula regarding physician-industry relationships in undergraduate, graduate and continuing medical education in Japan.

Involvement in pharmaceutical promotional activities is widespread among practicing physicians in Japan. The extent of such involvement varies according to certain physician characteristics. As a group, they are at risk for influence by pharmaceutical promotional activities. It may be necessary to completely ban not only receiving gifts but also meetings with PRs to maximally limit physician involvement in promotional activities, which may lead to more skeptical attitudes toward promotional activities and evidence-based prescribing behaviors.

## Supporting Information

Table S1 Multivariate predictors of physician involvement in promotional activities.
(0.05 MB DOC)

File S1 Survey questionnaire.
(0.06 MB DOC)

File S2 Result of factor analysis.
(0.04 MB DOC)

File S3 Calculations of a summary score for attitudinal scales.
(0.03 MB DOC)
