# Supplementary material for: Japanese Practicing Physicians' Relationships with Pharmaceutical Representatives: A National Survey
Source: PLoS One. 2010 Aug 13;5(8):e12193. doi: 10.1371/journal.pone.0012193 (PMC2921334; doi:10.1371/journal.pone.0012193)
Supplement: Table S1 — Multivariate predictors of physician involvement in promotional activities. (0.05 MB DOC) [file pone.0012193.s001.doc]

**Table S1. Multivariate predictors of physician involvement in promotional activities**.

| Characteristic | Odds ratio (95% confidence interval) | | | | | | |
| --- | --- | --- | --- | --- | --- | --- | --- |
|  | Meetings with PRs | Types of gifts or promotional events | | | | | |
|  |  | Drug samples | Stationery | Meals outside the workplace | Industry-sponsored CME events at the workplace | Industry-sponsored CME events outside the workplace | Financial subsidies to attend CME events |
| Sex | | | | | | | |
| Male | 1.00 | 1.00 | 1.00 | 1.00 | 1.00 | 1.00 | 1.00 |
| Female | 0.38(0.14-1.07) | 1.06(0.69-1.64) | 1.34(0.66-2.73) | 0.56(0.42-0.75)* | 1.96(1.30-2.96)* | 1.77(0.97-3.22) | 0.88(0.66-1.17) |
| Years in practice | | | | | | | |
| 1-20 years | 1.00 | 1.00 | 1.00 | 1.00 | 1.00 | 1.00 | 1.00 |
| 21 years ore more | 0.58(0.19-1.75) | 0.33(0.23-0.48)* | 0.64(0.33-1.22) | 0.55(0.42-0.72)* | 0.76(0.55-1.04) | 1.08(0.64-1.82) | 0.96(0.75-1.25) |
| Specialty | | | | | | | |
| Internal Medicine | 1.00 | 1.00 | 1.00 | 1.00 | 1.00 | 1.00 | 1.00 |
| General surgery | 4.25(0.48-37.76) | 0.45(0.25-0.79) | 1.13(0.40-3.23) | 0.89(0.59-1.36) | 0.54(0.31-0.95) | 0.86(0.37-2.02) | 0.68(0.45-1.02) |
| Orthopedic surgery | 0.51(0.15-1.72) | 1.00(0.52-1.92) | 1.86(0.54-6.34) | 1.53(1.00-2.36) | 0.55(0.32-0.96) | 1.15(0.46-2.86) | 1.14(0.75-1.73) |
| Pediatrics | 6.84(0.77-60.87) | 1.79(0.89-3.58) | 2.14(0.63-7.29) | 0.91(0.61-1.36) | 0.77(0.44-1.36) | 1.16(0.47-2.89) | 0.90(0.61-1.33) |
| Obstetrics-gynecology | 1.29(0.35-4.77) | 0.61(0.34-1.09) | 0.51(0.21-1.25) | 0.65(0.43-0.98) | 0.37(0.22-0.63)** | 0.53(0.24-1.17) | 0.34(0.23-0.51) ** |
| Psychiatry | 1.73(0.39-7.56) | 0.27(0.16-0.47)** | 0.53(0.21-1.31) | 1.15(0.76-1.73) | 0.49(0.29-0.85) | 0.86(0.37-2.00) | 1.13(0.76-1.69) |
| Ophthalmology | 5.48(0.62-48.38) | 1.01(0.52-1.94) | 0.94(0.34-2.60) | 0.61(0.41-0.92) | 0.44(0.26-0.76) | 0.51(0.24-1.11) | 0.31(0.21-0.47) ** |
| Practice setting | | | | | | | |
| Office | 1.00 | 1.00 | 1.00 | 1.00 | 1.00 | 1.00 | 1.00 |
| Hospital | 0.70(0.22-2.29) | 0.92(0.60-1.42) | 0.58(0.29-1.18) | 0.60(0.45-0.80)* | 1.62(1.11-2.37)* | 0.79(0.45-1.38) | 1.20(0.90-1.61) |
| Learning physician-industry relationships | | | | | | | |
| Yes | 1.00 | 1.00 | 1.00 | 1.00 | 1.00 | 1.00 | 1.00 |
| No | 1.68(0.65-4.38) | 1.36(0.94-1.95) | 1.49(0.81-2.72) | 1.28(0.97-1.69) | 1.05(0.75-1.46) | 1.26(0.74-2.14) | 1.02(0.78-1.34) |
| Learning critical appraisal skills | | | | | | | |
| Yes | 1.00 | 1.00 | 1.00 | 1.00 | 1.00 | 1.00 | 1.00 |
| No | 1.27(0.50-3.20) | 1.06(0.76-1.49) | 1.16(0.65-2.06) | 1.00(0.78-1.28) | 1.11(0.82-1.49) | 0.70(0.44-1.13) | 0.96(0.76-1.22) |
| Rules | | | | | | | |
| No rules | 1.00 | 1.00 | 1.00 | 1.00 | 1.00 | 1.00 | 1.00 |
| Banning meetings with PRs, not gifts | 0.29(0.06-1.50) | 0.61(0.28-1.33) | 0.69(0.19-2.48) | 0.94(0.53-1.68) | 0.60(0.30-1.22) | 1.15(0.34-3.95) | 0.84(0.47-1.51) |
| Banning gifts, not meetings with PRs | 0.65(0.18-2.35) | 0.72(0.44-1.17) | 0.65(0.31-1.36) | 0.31(0.22-0.45)* | 1.06(0.66-1.69) | 0.94(0.49-1.81) | 0.55(0.39-0.77) * |
| Banning both meetings with PRs and gifts | 0.14(0.04-0.47)* | 0.35(0.17-0.70)* | 0.29(0.11-0.72)* | 0.21(0.11-0.41)* | 0.68(0.34-1.37) | 0.39(0.17-0.86)* | 0.46(0.26-0.82) * |

Abbreviations: PR, pharmaceutical representative; CME, continuing medical education

+ We carried out pairwise comparisons of each specialty with every other specialty although only the comparison of the other specialties with internal medicine was shown because of limited space.

* *P*-value less than 0.05 for the comparison with reference group (not adjusted for multiple comparisons)

** *P* value of less than 0.0017 for the comparisons between specialties (adjusted for multiple comparisons)
