# Supplementary material for: Japanese Practicing Physicians' Relationships with Pharmaceutical Representatives: A National Survey
Source: PLoS One. 2010 Aug 13;5(8):e12193. doi: 10.1371/journal.pone.0012193 (PMC2921334; doi:10.1371/journal.pone.0012193)
Supplement: File S1 — Survey questionnaire. (0.06 MB DOC) [file pone.0012193.s002.doc]

**File S1. Survey questionnaire.**

1. On average, how often do you meet pharmaceutical representatives (PRs)?

| Never | once a month or less | Twice or three times a month | Once a week | Twice or three times a week | Nearly everyday |
| --- | --- | --- | --- | --- | --- |

1. On, average, how often do you receive or participate in the following gifts or events?
2. Drug samples

| Never | once a month or less | Twice or three times a month | Once a week | Twice or three times a week | Nearly everyday |
| --- | --- | --- | --- | --- | --- |

1. Stationery such as pens and notepads

| Never | once a month or less | Twice or three times a month | Once a week | Twice or three times a week | Nearly everyday |
| --- | --- | --- | --- | --- | --- |

1. Industry-sponsored CME events inside the workplace

| Never | once a month or less | Twice or three times a month | Once a week | Twice or three times a week | Nearly everyday |
| --- | --- | --- | --- | --- | --- |

1. Meals outside the workplace

| Never | once a month or less | Twice or three times a month | Once a week | Twice or three times a week | Nearly everyday |
| --- | --- | --- | --- | --- | --- |

1. Industry-sponsored CME events outside the workplace

| Never | once a month or less | Twice or three times a month | Once a week | Twice or three times a week | Nearly everyday |
| --- | --- | --- | --- | --- | --- |

1. Financial subsidies to attend CME events

| Never | once a month or less | Twice or three times a month | Once a week | Twice or three times a week | Nearly everyday |
| --- | --- | --- | --- | --- | --- |

1. To what extent do you agree/disagree the following statements regarding relationships with pharmaceutical representatives?

a. PRs play an important role in CME for practicing physicians.

| Agree | Somewhat agree | Neutral | Somewhat disagree | Disagree |
| --- | --- | --- | --- | --- |

b. PRs provide accurate information about new medications.

| Agree | Somewhat agree | Neutral | Somewhat disagree | Disagree |
| --- | --- | --- | --- | --- |

c. PRs provide accurate information about old (established) medications.

| Agree | Somewhat agree | Neutral | Somewhat disagree | Disagree |
| --- | --- | --- | --- | --- |

d. Discussions with PRs have an unfavorable impact on my prescribing behaviors.

| Agree | Somewhat agree | Neutral | Somewhat disagree | Disagree |
| --- | --- | --- | --- | --- |

e. Gifts from PRs have an unfavorable impact on my prescribing behaviors, regardless of the monetary value

| Agree | Somewhat agree | Neutral | Somewhat disagree | Disagree |
| --- | --- | --- | --- | --- |

f. Gifts from PRs have an unfavorable impact on other physicians’ prescribing behaviors, regardless of the monetary value

| Agree | Somewhat agree | Neutral | Somewhat disagree | Disagree |
| --- | --- | --- | --- | --- |

g. It is appropriate to receive gifts of low monetary value from PRs

| Agree | Somewhat agree | Neutral | Somewhat disagree | Disagree |
| --- | --- | --- | --- | --- |

h. It is appropriate to receive gifts of high monetary value from PRs.

| Agree | Somewhat agree | Neutral | Somewhat disagree | Disagree |
| --- | --- | --- | --- | --- |

4. To what extent have you had an opportunity to learn physician-pharmaceutical industry relationships?

| None | Little | A little | Some | Substantial |
| --- | --- | --- | --- | --- |

1. To what extent have you had an opportunity to learn critical appraisal skills of the medical literature about treatment?

| None | Little | A little | Some | Substantial |
| --- | --- | --- | --- | --- |

6.　Which of the followings do you do first most commonly when a new medication becomes available?

1. Seek published findings for its effectiveness
2. Ask colleagues/specialists for their opinions
3. Collect information from pharmaceutical representatives
4. Use on a few patients and monitor

7. Which of the followings do you do most commonly when faced with a patient who expects a prescription which is not clinically indicated?

1. Agree readily without explaining it is not clinically indicated
2. Explain it is not clinically indicated, but prescribe reluctantly
3. Explain it is not clinically indicated, and not prescribe

8. How satisfactory/unsatisfactory do you feel about a patient consultation that ends with advice only (without prescribing any medications)?

1. Satisfactory
2. Somewhat satisfactory
3. Somewhat unsatisfactory
4. Unsatisfactory

9. When generic options are available, which of generic or brand-name medications do you think you should prescribe?

1. Prescribe generic medications
2. Neutral
3. Prescribe brand-name medications

10.　Are there rules banning gifts from PRs and/or meetings with PRs in your current workplace?

1. There are rules banning gifts from PRs, but not meetings with PRs
2. There are rules banning meetings with PRs, but not gifts from PRs
3. There are rules banning both gifts from and meetings with PRs
4. There are no rules banning both gifts from and meetings with PRs

11.　Provide your background information under the following headings.

Sex　　　　（　　）Male （　　）Female

No. of years in practice

（　　）0～5 yr. （　　）6～10 yr （　　）11～15yr

（　　）16～20 yr　 （　　）21～30yr （　　）31 yr or more

Specialty

（　 ）Internal Medicine （　 ）Surgery （　 ）Orthopedics （ 　 ）Pediatrics （　　）Obstetrics/gynecology　　 （　　）Psychiatry　 （　　）Ophthalmology

（　　）Other [ ]

Practice setting

1. Office

2. Hospital

3. Other　[ ]
