# Supplementary material for: Japanese Practicing Physicians' Relationships with Pharmaceutical Representatives: A National Survey
Source: PLoS One. 2010 Aug 13;5(8):e12193. doi: 10.1371/journal.pone.0012193 (PMC2921334; doi:10.1371/journal.pone.0012193)
Supplement: File S2 — Result of factor analysis. (0.04 MB DOC) [file pone.0012193.s003.doc]

**File S2. Results of factor analysis**

Pattern matrix after rotation*

| Statement | Component | | |
| --- | --- | --- | --- |
|  | Informational Value | Immunity | Appropriateness |
| a. PRs play an important role in CME | .570 | -.064 | .081 |
| b. PRs provide accurate information about new medications | .892 | -.005 | -.019 |
| c. PRs provide accurate information about old (established) medications | .697 | -.013 | -.031 |
| d. Discussions with PRs have unfavorable impact on my prescribing behaviors | -.085 | .514 | .117 |
| e. Gifts from PRs have unfavorable impact on my prescribing behaviors | .073 | .963 | .006 |
| f. Gifts from PRs have unfavorable impact on other physicians’ prescribing behaviors | -.049 | .675 | -.078 |
| g. A low-value gift from PRs is appropriate | .008 | -.017 | .794 |
| h. A high-value gift from PRs is appropriate | .012 | .058 | .624 |

Abbreviations: PR, pharmaceutical representative; CME, continuing medical education.

* Extraction method: principal axis factoring. Rotation method: Promax with Kaiser Normalization

Factor correlation matrix**

| Factor | Informational Value | Immunity | Appropriateness |
| --- | --- | --- | --- |
| Informational value | 1.000 | -.165 | .128 |
| Immunity | -.165 | 1.000 | -.128 |
| Appropriateness | .128 | -.128 | 1.000 |

** Principal component analysis, Promax rotation with Kaiser normalization, minimum eigenvalue less than 1
