# Supplementary material for: Japanese Practicing Physicians' Relationships with Pharmaceutical Representatives: A National Survey
Source: PLoS One. 2010 Aug 13;5(8):e12193. doi: 10.1371/journal.pone.0012193 (PMC2921334; doi:10.1371/journal.pone.0012193)
Supplement: File S3 — Calculations of a summary score for attitudinal scales. (0.03 MB DOC) [file pone.0012193.s004.doc]

**File S3. Calculations of a summary score for attitudinal scales.**

For items of Informational Value Score, agree was scored 5, somewhat agree 4, neutral 3, somewhat disagree 2, and disagree 1.The scores of 3 items were summed, subtracted by the minimum score 3, then divided by 12 (the maximum score 15 subtracted by the minimum score 3). For items of Appropriateness Score, agree was scored 5, somewhat agree 4, neutral 3, somewhat disagree 2, and disagree 1. The scores of two items were summed, subtracted by the minimum score 2, then divided by 8 (the maximum score 10 subtracted by the minimum score 2). For items of Immunity Score, agree was scored 1, somewhat agree 2, neutral 3, somewhat disagree 4, and disagree 5. The scores of 3 items were summed, subtracted by the minimum score 3, then divided by 12 (the maximum score 15 subtracted by the minimum score 3).

The ranges of the three scores were from 0 to 1.0. The score of 0 was interpreted as the physicians’ perception about PRs being minimal informational value, themselves as minimally immune against promotion, or gifts being as totally inappropriate.
